# The complete mitochondrial genome of the New Zealand parasitic roundworm *Teladorsagia circumcincta* (Trichostrongyloidea: Haemonchidae) field strain NZ_Teci_NP

**DOI:** 10.1080/23802359.2019.1660241

**Published:** 2019-09-06

**Authors:** Nikola Palevich, Paul H. Maclean, Makedonka Mitreva, Richard Scott, David Leathwick

**Affiliations:** a AgResearch Ltd., Grasslands Research Centre, Palmerston North, New Zealand;; b McDonnell Genome Institute and Department of Medicine, Washington University School of Medicine, Saint Louis, Missouri, United States of America

**Keywords:** *Teladorsagia circumcincta*, roundworm, gastrointestinal, trichostrongyloid, nematode, phylogeny

## Abstract

The complete mitochondrial genome of the New Zealand parasitic nematode *Teladorsagia circumcincta* field strain NZ_Teci_NP was sequenced and annotated. The 14,083 bp-long mitogenome contains 12 protein-coding genes (atp8 gene missing), two ribosomal RNAs (rRNAs), and 22 transfer RNAs (tRNAs). Phylogenetic analysis showed that *T. circumcincta* NZ_Teci_NP forms a monophyletic cluster with the remaining Haemonchidae species and further reinforces the high levels of diversity and gene flow observed among Trichostrongylidae.

The large and highly variable mitochondrial (mt) genomes of helminths (worms), including parasitic nematodes (roundworms), are ideal sources of molecular markers suitable for studying population genetic structures and evolution. *Teladorsagia circumcincta* NZ_Teci_NP was selected for genome sequencing as a representative of an anthelmintic-susceptible NZ field strain of *T. circumcincta*. The specimen was collected from the Palmerston North area (40°21.3′S, 175°36.7′E) and is stored (accession number: NPX120886) and available upon request from AgResearch Ltd., Grasslands Research Centre. High molecular weight genomic DNA was isolated from multiple *T. circumcincta* adult males using a modified phenol:chloroform protocol (Palevich et al. 2017; Palevich, Kelly, et al. 2019). The Illumina MiSeq (Macrogen, Korea) platform was used to amplify the entire mitochondrial genome sequence (GenBank accession number: MN013406).

The mitogenome of NZ_Teci_NP (14,083 bp) is standard in size and comparable to the *T. circumcincta* (GQ888720) strain (Jex et al. 2010; Choi et al. 2017; Palevich, Maclean, et al. 2019). For example, all genes are transcribed in the same direction, there is a lack of the Atp8 gene, it contains 12 protein-coding genes (PCGs), two rRNAs, and 22 tRNAs. All 12 PCGs use standard ATN/TAN start/stop codons, respectively. The studied genome has a high T content (46.1%) and a low C content (7.2%), resulting in a very strong A + T bias (77.3%). Gene order, sizes, and all common organization features are relatively conserved among the 43 nematode mitogenomes (usually 13.6–14.3 kb) (Jex et al. 2009; Palevich et al. 2018; Palevich, Maclean, Baten et al. 2019; Palevich, Maclean, et al. 2019).

The phylogenetic position of *T. circumcincta* was estimated using maximum-likelihood, implemented in RAxML version 8.2.11 (1000 bootstrap replications) (Stamatakis 2014), and the Bayesian inference (BI), implemented in MrBayes version 3.2.6 (default settings, four MCMC chains, 6.34–106 generations) (Huelsenbeck and Ronquist 2001) approaches. Mitogenome sequences of all 36 available nematode species were retrieved from GenBank. Analyses were performed both on the entire nucleotide sequences of the complete mitogenomes and using only the concatenated mitochondrial PCGs and rRNA genes, producing identical dendrogram topologies (Figure 1). *Teladorsagia circumcincta* formed a monophyletic cluster with the remaining Trichostrongylidae species, which then formed a sister clade with the Strongylidae family. Overall, the dendrogram topology is highly congruent with recent results (Palevich, Maclean, et al. 2019). In the pursuit of improving the phylogenetic resolution within the phylum Nematoda, future efforts should focus on the availability of more complete mitogenomes across all nematode species, and especially for different strains/isolates.

**Figure 1.**
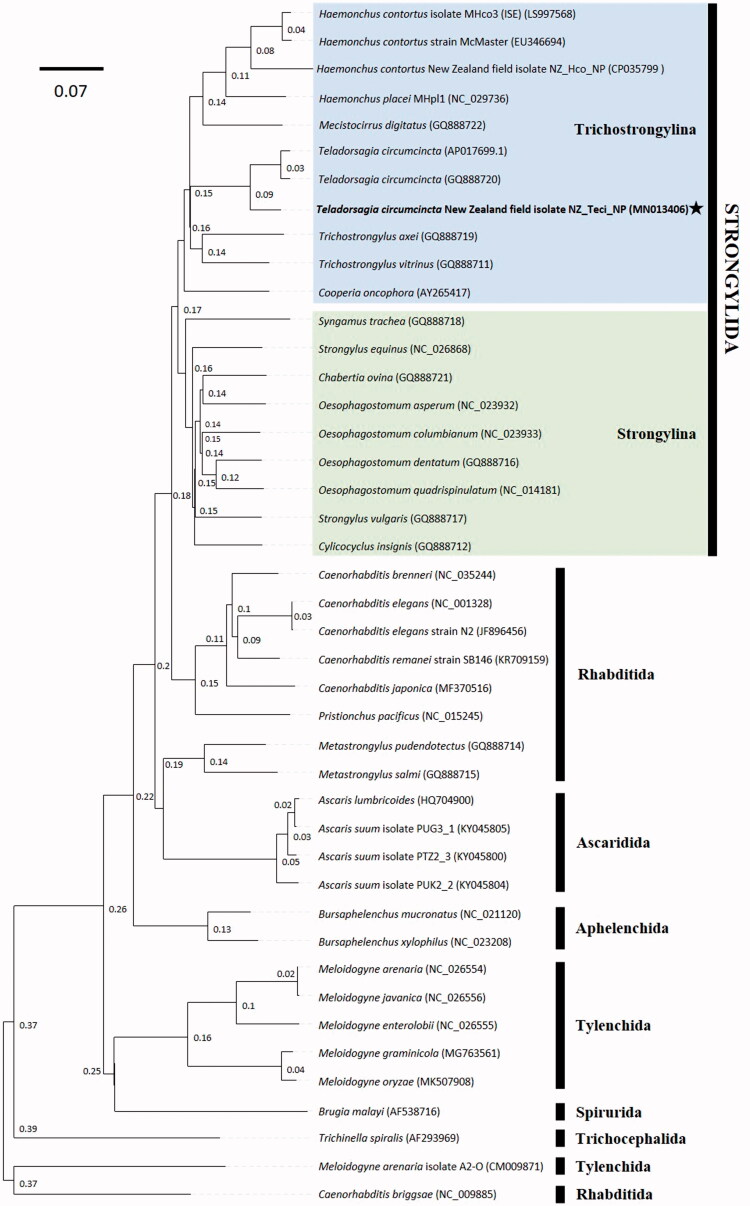
Phylogenetic analysis of the complete mt genomes for all 43 nematode species or isolates available in GenBank. The evolutionary relationships between the *T. circumcincta* field strain NZ_Teci_NP (highlighted by a black star) and the two major suborders of the Strongylida are represented by coloured boxes (Trichostrongylina (blue) and Strongylina (green)). Species representing the Rhabditida, Ascaridida, Aphelenchida, Tylenchida, Spirurida, and Trichocephalida have been included as outgroups. Phylogenetic analysis was conducted using maximum-likelihood and Bayesian inference (MrBayes). The numbers above the midpoint of each tree branch represent the statistical support for each node (based on posterior probability score). The phylogram provided is presented to scale (scale bar = 0.07 estimated number of substitutions per site) and GenBank accession numbers are provided (in parentheses) for all reference sequences. An identical topology was found with maximum-likelihood; all nodes were supported by >99% bootstrap re-sampling (*n* = 1000).

## References

[CIT0001] Choi YJ, Bisset SA, Doyle SR, Hallsworth-Pepin K, Martin J, Grant WN, Mitreva M 2017 Genomic introgression mapping of field-derived multiple-anthelmintic resistance in *Teladorsagia circumcincta*. PLoS Genet. 13:e1006857. 28644839 10.1371/journal.pgen.1006857 PMC5507320

[CIT0002] Huelsenbeck JP, Ronquist F 2001 MRBAYES: Bayesian inference of phylogenetic trees. Bioinformatics. 17:754–755. 11524383 10.1093/bioinformatics/17.8.754

[CIT0003] Jex AR, Hall RS, Littlewood DTJ, Gasser RB 2010 An integrated pipeline for next-generation sequencing and annotation of mitochondrial genomes. Nucleic Acids Res. 38:522–533. 19892826 10.1093/nar/gkp883 PMC2811008

[CIT0004] Palevich N, Britton C, Kamenetzky L, Mitreva M, de Moraes Mourão M, Bennuru S, Quack T, Scholte LLS, Tyagi R, Slatko BE 2018 Tackling hypotheticals in helminth genomes. Trends Parasitol. 34:179–183. 29249363 10.1016/j.pt.2017.11.007 PMC11021132

[CIT0005] Palevich N, Maclean P, Baten A, Scott A, Leathwick D 2019 The complete mitochondrial genome of the New Zealand parasitic roundworm *Haemonchus contortus* (Trichostrongyloidea: Haemonchidae) field strain NZ_Hco_NP. Mitochondrial DNA B. 2208. 10.1080/23802359.2019.1624634 PMC7687515 33365477

[CIT0006] Palevich N, Kelly WJ, Ganesh S, Rakonjac J, Attwood GT 2019 *Butyrivibrio hungatei* MB2003 Competes Effectively for Soluble Sugars Released by *Butyrivibrio proteoclasticus* B316^T^ during Growth on Xylan or Pectin. Appl Environ Microbiol. 85:e02056–02018. 30478228 10.1128/AEM.02056-18 PMC6344614

[CIT0009] Palevich N, Maclean PH, Baten A, Scott RW, Leathwick DM 2019 The Genome Sequence of the Anthelmintic-Susceptible New Zealand Haemonchus contortus. Genome Biol Evol. 11:1965–1970. doi:10.1093/gbe/evz141 31263885 PMC6644846

[CIT0007] Palevich N, Kelly WJ, Leahy SC, Altermann E, Rakonjac J, Attwood GT 2017 The complete genome sequence of the rumen bacterium *Butyrivibrio hungatei* MB2003. Stand Genomic Sci. 12:72. 29225728 10.1186/s40793-017-0285-8 PMC5716241

[CIT0008] Stamatakis A 2014 RAxML version 8: a tool for phylogenetic analysis and post-analysis of large phylogenies. Bioinformatics. 30:1312–1313. 24451623 10.1093/bioinformatics/btu033 PMC3998144

